# Features Linked to Treatment Outcomes in Behavioral Addictions and Related Disorders

**DOI:** 10.3390/ijerph20042873

**Published:** 2023-02-07

**Authors:** Gemma Mestre-Bach, Marc N. Potenza

**Affiliations:** 1 Facultad de Ciencias de la Salud, Universidad Internacional de La Rioja, 26006 La Rioja, Spain; 2 Department of Psychiatry, Yale University School of Medicine, New Haven, CT 06510, USA; 3 Connecticut Mental Health Center, New Haven, CT 06519, USA; 4 Connecticut Council on Problem Gambling, Wethersfield, CT 06109, USA; 5 Wu Tsai Institute, Yale University, New Haven, CT 06510, USA; 6 Yale Child Study Center, Yale University School of Medicine, New Haven, CT 06510, USA; 7 Department of Neuroscience, Yale University School of Medicine, New Haven, CT 06510, USA

**Keywords:** behavioral addictions, gambling disorder, relapse, dropout, treatment outcome

## Abstract

Behavioral addictions are incompletely understood with respect to their underlying etiologies. This incomplete understanding may contribute to the frequent relapse and dropout rate often observed with behavioral addictions. The present state-of-the-art review aimed to review the literature that explored sociodemographic and clinical factors that link to poor treatment responses. Despite multiple studies, the definitions and evaluations of relapse and dropout are heterogeneous, complicating comparisons across studies. A scientific consensus on the conceptualization of both terms would help to better understand psychological features linked to treatment outcomes in behavioral addictions.

## 1. Introduction

Gambling disorder (GD), (internet) gaming disorder (IGD), compulsive buying–shopping disorder (CBSD), and compulsive sexual behavior disorder (CSBD) have been recognized as mental disorders by the Diagnostic and Statistical Manual of Mental Disorders - Fifth Edition (DSM-5) [1] or the International Classification of Diseases - 11th Revision (ICD-11) to varying degrees, with some (e.g., CBSD) that may be considered as an “other specified disorder” in the ICD-11 nomenclature system [2,3]. All have been proposed to be behavioral addictions, although some conditions remain more debated than others [4,5,6]. Mechanistically, these behaviors and conditions are incompletely understood, especially with respect to their etiologies. However, all involve short-term rewards that may promote the maintenance of these behaviors despite being associated with negative consequences. Additionally, there typically exist notable alterations in control over the behaviors, and all show some shared (as well as unique) features with respect to substance use disorders (SUDs) [7].

Despite an incomplete understanding of the etiologies, numerous pharmacological and psychological therapies have been tested and proposed to treat behavioral addictions, especially cognitive behavioral therapy (CBT) [8,9,10,11]. However, there still remains controversy about how best to define recovery, as well as factors associated with treatment outcomes, such as dropout and relapse. Such controversies exist for SUDs and behavioral addictions, with recent definitions from national organizations regarding how best to define recovery having been published, for example, for alcohol use disorder [12].

Regarding recovery, difficulties in conceptualization may derive from differences in defining therapeutic objectives of treating behavioral addictions and, consequently, in defining the concepts of abstinence or controlled behavior (e.g., responsible gambling) [13,14]. In the case of controlled behavior, moreover, its evaluation may involve assessments of pre-treatment changes, and it may be complex to assess modifications in levels/severities of addictive behaviors [13]. Although some complex biopsychosocial theoretical models attempted to establish definitions of recovery (all tend to agree that recovery may be associated with a reduction in the negative consequences associated with the addictive behaviors), they each tended to focus on different indicators of success, depending on, for example, whether they are examined from public health, psychological, or social domains [13]. Some authors considered recovery as qualitative changes in diagnostic status at follow-up that demonstrates improvements in mental health, reductions in severities of addictive behaviors, and reductions in frequencies of addictive behaviors [15]. Other attempts to define and assess recovery, such as the development of the Recovery Index for Gambling Disorder (RIGS), contemplated other dimensions, related not only to reductions in addictive behaviors, but also improvements in urge coping, recovery wisdom, life functioning, interpersonal relationships, and mental health [16].

Considering the response to treatment, some authors [17] suggested that high rates of dropout and relapse in behavioral addictions may reflect ambivalence presented by individuals with these disorders about modifying addictive behaviors that may be rewarding in the shorter term, but associated with multiple negative consequences in the longer term. Therefore, the combination of reinforcers and punishing consequences of behavioral addictions may generate approach–avoidance conflicts, as well as motivational ambivalences, which may interfere with adherence to treatment and lead to both dropout and relapse [17].

Due to complexities in categorizing recovery and treatment outcomes, the present state-of-the-art review had as its main objective a review of the literature focused on sociodemographic and clinical factors associated with dropout and relapse in the case of behavioral addictions. Compiling this information should help advance current intervention development by laying a foundation for theory-driven prevention or interventions approaches as etiologies become better understood.

## 2. Materials and Methods

This state-of-the-art review aimed to provide a comprehensive narrative synthesis of existing studies about psychological features linked to treatment outcomes. PubMed and Google Scholar were used to search the scientific literature that had been published in peer-reviewed international journals up to 28 December 2022. Original studies with human samples involving one or more participants were considered, as were reviews. Articles in English or Spanish focusing on GD, IGD, CBSD, and CSBD were considered, and gray literature was excluded. Multiple searches using exclusively English terms were conducted. An example of search keywords is: (gambling OR gaming OR “compulsive sexual behavior disorder” OR buying) AND (“treatment outcome” OR “intervention outcome” OR “treatment response” OR “intervention response” OR dropout OR relapse OR adherence). Articles were not filtered according to a specific time range; all articles that met the inclusion criteria were included, regardless of the year of publication. A first search was made taking into account title and abstract, and then the identified articles were read in their entirety by one researcher.

## 3. Results

A listing of main factors considered in the present review is tabulated below (Table 1).

### 3.1. Dropout

Data provide insights into the presence of dropouts across interventions for different behavioral addictions. However, there is no consensus on how best to categorize dropouts. Usually, a dropout is described as leaving an intervention before it ends, although there is heterogeneity in definitions. The conceptualization of dropout should consider whether an intervention has a pre-established number of sessions or not [18]. If so, multiple studies have considered dropout to be nonattendance at a specific number of sessions (which varies among studies), while, if not, dropout is considered according to the therapist’s judgment of appropriate termination [18]. This lack of homogeneity makes comparison between studies difficult. For example, a systematic review and meta-analysis on dropout in GD found that dropout rates in GD were significantly higher when dropout was defined as attending all treatment sessions, as compared to defining it as attending a pre-specified number of sessions other than the total number of sessions established in the treatment protocol, as well as when it was defined as the therapist’s judgement [19].

In addition, dropout may occur at multiple stages of treatment: (a) pre-treatment dropout occurs when an individual drops out before starting the intervention; (b) within-treatment dropout occurs when an individual leaves the treatment following initiation but before completing it; and (c) follow-up dropout occurs following treatment but before completing the follow-up assessments [18]. Moreover, pre-treatment dropout often involves non-provision of consent to initiate an intervention, and the terms “dropout”, “premature termination”, and “attrition” are often used interchangeably [18]. Dropout appears higher during GD treatment onset (the first sessions-two months) and appears to decrease thereafter [20,21].

#### 3.1.1. Sociodemographic Features and Dropout

Age. Possible links between age and dropout in behavioral addictions has been examined little [18,22]. Moreover, findings appear somewhat contradictory. On the one hand, some authors reported associations between older age and dropout [23]. Some authors highlighted older age as a significant statistical predictor of dropout in the case of individuals with GD [23]. It should be noted that these authors considered older age to be all those individuals aged 26+ years, as compared to those younger than 26. However, there is no consensus on the conceptualization of the term “older age”, since other studies considered other age ranges, such as 31–70 [24] or 50–90 [25] years old.

On the other hand, others noted that younger participants with GD exhibited higher dropout [26,27], and attended fewer treatment sessions before abandoning interventions [27]. Young age has even been considered in different studies as a main predictor of dropout from GD treatment [22,28]. Some studies differentiating pre-treatment from in-treatment dropout observed that younger age was associated with higher pre-treatment dropout [29]. Some authors [20] reported that older female patients attended a greater number of CBT sessions during treatment for GD.

Gender. Associations between gender and dropout in treatments for behavioral addictions need to be further examined. On the one hand, as highlighted in a review [18], different studies have not observed a clear link between both factors in GD [30,31,32,33,34,35]. On the other hand, some studies noted that all non-completers in a residential treatment for GD were male [26]. Finally, other studies observed that female gender was associated with higher treatment dropout rates for GD [36,37]. Therefore, some authors highlighted that being female may be a vulnerability factor for poor treatment outcome [36].

Education level/years of education. Only a few studies examined education and dropout. While some highlighted an association between lower education level and higher dropout in GD treatment [28], others found no such relationship [38]. Finally, other studies found that high levels of education statistically predict dropout [39]. Therefore, links between education level and dropout warrant more examination.

Employment status. Associations between employment status and dropout have been seemingly contradictory, as previously indicated [18]. For example, some authors reported that not having full-time employment is a significant predictor of dropout in GD treatment [38]. Similarly, another study observed that a higher probability of dropout in the group of patients with GD and sports betting was associated with being unemployed [37]. This could reflect the fact that the lack of economic stability may increase stress experienced and, consequently, interfere with responses to treatment [18]. In contrast, other studies did not find associations between employment and dropout in individuals with GD who attended Gam-Anon (a self-help group to support family members (or other affected individuals) of people with GD) [40].

Income/social status. Studies did not find clear associations between income and dropouts in GD treatment [30,33,40]. However, some observed that the likelihood of dropout in individuals with GD and without sport betting is higher in those with lower social status [37].

Civil status and social support. Single marital status appears to be one of the most significant predictors of dropout in GD treatment [22], although other studies have not found a significant relationship between civil status and dropout in GD [38]. In GD, it has been reported that poor family support has been closely associated with dropout [36]. Considering the participation of spouses of individuals with GD in treatment sessions, Brown [40] noted that of the dropout group, fewer spouses had attended Gam-Anon sessions. In addition, none of the spouses of the individuals in the dropout group were still attending sessions at the time dropout occurred. The extent to which social support is linked to dropout in behavioral addictions other than GD warrants more direct investigation.

#### 3.1.2. Co-Occurring Disorders and Dropout

Psychopathological distress. In GD, psychopathology and treatment outcome have been linked. For example, women with GD with more (versus less) psychopathological distress had more frequent dropout [20]. These results may reflect the fact that the presence of psychopathology may be associated with, among other factors, greater severity of GD and higher levels of impulsivity, greater gambling urges, more cognitive distortions, and greater psychosocial difficulties, and all of these factors may increase risk of therapeutic abandonment, as has been previously suggested [20].

Suicidality and co-occurring behaviors/disorders. Among individuals with GD, those with and without suicidal ideation/attempts did not differ in dropout rates [36]. Another factor that has been considered with respect to dropout is use of tobacco and substance use [29]. It has been suggested that substance use may be linked with pre-treatment dropout, while tobacco use may be associated with in-treatment dropout [29]. However, other studies have not identified an association between substance use/abuse and treatment dropout [41]. SUDs, along with post-traumatic stress and anti-social personality disorders, may link to early dropout in GD [42]. Likewise, higher levels of obsessive–compulsive symptomatology may predict dropout [43]. Higher depression levels have also been associated with dropout [23].

#### 3.1.3. Personality Features and Dropout

There may exist associations between certain personality features and dropout. An association between low conscientiousness and dropout has been reported in GD treatment [44,45]. Conscientiousness has been understood as a “will to achieve”, so individuals with low conscientiousness may be less diligent and achieving and may have difficulties controlling impulsive behaviors [45]. Therefore, individuals with GD and low conscientiousness may have greater difficulty managing GD symptomatology and adhering to treatment. Low agreeableness also appears to be a significant predictor of dropout in GD treatment [44].

Other personality dimensions have been associated with treatment dropout. Novelty-seeking has been associated with dropout from GD treatment [36]. Some authors have suggested novelty-seeking, along with suicidal behavior, as a main predictor of dropout from GD treatment [36]. Low self-directedness [36], cooperativeness [36], and lack of perseverance [46] also seem linked to dropout from GD treatment.

#### 3.1.4. Impulsivity, Executive Functioning, and Dropout

Several studies explored possible associations between impulsivity and dropout. Some authors highlight that high impulsivity [26,47] and sensation-seeking are associated with dropout [41]. In fact, sensation-seeking features have been identified as a significant statistical predictor of dropout from GD treatment [48]. More specifically, sensation-seeking may predict both shorter-term and 24 month dropout in GD [49]. Affect-driven impulsivity features have been linked to GD treatment dropout [50], as have high impulsivity and exploratory excitability [51]. Elevated levels of reward sensitivity have also been associated with an increased likelihood of dropout in women with GD or CBSD [52]. Regarding executive functioning, poor cognitive flexibility has been associated with end-of-GD-treatment dropout [53].

#### 3.1.5. Addiction-Related Features, Disorder Severity, and Dropout

Disorder severity. When evaluating associations between disorder severity and dropout, seemingly contradictory results have been reported. For example, some authors proposed lower GD severity as a predictor of treatment success [54]. However, some studies linked (in-)treatment dropout and lower severity of GD [20,29]. The authors hypothesized that these findings may be due to the fact that women with lower GD severity may experience less GD-related interference in their daily lives and, therefore, perceive the disorder as more ego-syntonic, which may undermine treatment adherence [20]. Other authors, despite hypothesizing that those individuals with GD and suicidal ideation/attempts would have a worse response to treatment due to greater clinical severity, found no difference between the dropout rates of individuals with GD with or without suicidal ideation/attempts [36]. Finally, some researchers observed a negative but non-significant correlation between GD severity and therapy compliance [50] or a lack of direct association between GD severity and dropout [37]. Similarly, another study found no difference in treatment outcomes among patients with GD categorized according to DSM-5 severity levels [49]. Following these findings, the authors wondered whether the sum of diagnostic criteria used by the DSM-5 to measure severity reflected the complex reality of GD symptom severity and whether all diagnostic criteria have the same weight with respect to GD severity.

Disorder age of onset / disorder duration. No clear association between age of onset of GD and dropouts during treatment has been observed [41,55]. Similarly, no link between duration of GD and treatment dropout has been described [41].

Disorder subtypes/preferences. In studies of GD, some authors have found no differences in dropouts when gambling preferences are taken into account [36]. However, other studies have linked delay discounting to dropout in individuals with GD whose preferences are non-strategic or mixed types of gambling [27]. In another study, gambling on machines has been associated with pre-treatment dropout, while sports betting has been linked with dropout during GD treatment [56]. Another study identified online gambling and gambling on poker as being associated with dropout [23].

Other disorder-related features. In GD, distortions relating to predictive control, a greater tendency towards gambler’s fallacy, have been linked to dropout [26]. Likewise, the commission of gambling-related offenses with legal consequences has been associated with dropout [57].

#### 3.1.6. Other Factors and Dropout

Another factor studied in relation to dropout is emotional regulation. Among 459 patients, of whom 182 had GD with the rest having eating disorders, greater emotional dysregulation was associated with dropout [58].

Another factor associated with dropout has been motivation to change. It could be hypothesized that those patients with behavioral addictions who are less motivated to change might have more difficulties in identifying treatment goals and adhering to treatment [18]. However, empirical evidence in this regard is lacking. Motivation to change was explored in individuals with GD, but no association was found between it and dropout [32].

### 3.2. Relapse

The literature focusing on relapse in behavioral addictions is scarce. As with dropout, there are multiple and heterogeneous definitions and assessments of relapse. For example, relapse has been defined as any behavior (e.g., gambling in GD) that goes against the individual’s personal goals. For individuals who intend to be abstinent, any episode (of gambling) may imply a relapse. However, for those who intend to achieve controlled gambling, relapse may be defined differently [59]. Other definitions have included the concept of “loss of control” and consider that if there are no feelings of “loss of control”, the episode (e.g., gambling in GD) should not be considered a relapse [59]. Finally, other definitions, for example, have indicated that a relapse would include more than two episodes of gambling within 12 months of treatment [59]. In this vein, as some authors suggest [15], the diagnostic criteria for some of the behavioral addictions specify that for a diagnosis to be made, symptoms must have occurred within the last 12 months. However, the duration of follow-up in different studies is inadequate to effectively track the progression and potential re-emergence of addictive behaviors in behavioral addictions. Other complexities exist. For example, does gambling on the lottery constitute a relapse for someone with GD whose problematic gambling has involved solely electronic gambling machines? Analogously, how would one consider any purchase or sexual activity in people with CBSD and CSBD, respectively?

Relapse prevention models attempt to consider complexities of relapses in addictions. These theoretical proposals suggest that there is a set of relapse-precipitating factors, such as craving, that generate relapse if specific individual factors, such as coping skills, are insufficient, creating a self-perpetuating cycle [60]. In addition, it has been described that each relapse episode may involve complex sets of social and psychological behaviors in which multiple factors interact sequentially to generate in individuals with addictions series of behavior and mental events that usually end in relapse [61].

#### 3.2.1. Sociodemographic Features and Relapse

Age. An association between age and relapse has been described [62]. Some studies suggest that age and relapse have an inverse relationship [62], with younger age linked to relapse in individuals with GD [27].

Gender. Some studies associated female gender with relapse in individuals with GD [36,63].

Education level/years of education. Lower educational levels have been associated with relapse women with GD [20]. The authors suggested that this association may relate to insufficient skills necessary to modify maladaptive cognitive patterns in GD.

Motherhood. In CBSD treatment, being a mother has been linked with a lower likelihood of relapse [64]. Motherhood may be an internal facilitator for adherence to treatment, possibly stemming from desires to be good mothers and break cycles of addiction [64].

Civil status and social support. Poor family support and social deprivation have been associated with relapse in GD treatment [36,65]. Similarly, one of the most relevant predictors of relapse in GD seems to be single marital status [22]. Among patients with GD and sports betting, not being married is associated with a higher probability of relapse [37]. Other authors observed that relapses among women with GD during CBT treatment were more frequent in women who were divorced [20].

In assessing the involvement of spouses in the treatment of patients with GD, there is little evidence regarding effects on relapse [66]. Some studies observed that those patients whose spouses were involved in treatment sessions had lower likelihoods of relapse [67]. However, opposite results have been reported in other studies. For example, an increased likelihood of relapse during GD treatment was related to spousal or partner involvement in therapy sessions [28]. The authors concluded, therefore, that the incorporation of a family member in therapy sessions should be considered with caution, since it may have a negative effect on patients’ treatment responses.

#### 3.2.2. Co-Occurring Disorders and Relapse

Psychopathological distress. Psychological distress has been described as a precipitating factor for relapse in individuals with GD [68]. Relatedly, some authors have found that, in a group of individuals with GD, the probability of relapse was higher for those with greater psychopathology [37].

Co-occurring disorders. It has been observed that higher levels of obsessive–compulsive symptomatology may predict relapse [43]. Among individuals with GD, suicidal ideation/attempts were not linked to relapse [36]. Finally, among women with GD, the relapse was more frequent in those who used illegal substances and those without tobacco use [20].

#### 3.2.3. Personality Features and Relapse

Studies evaluating relationships between personality features and relapse have obtained seemingly contradictory results. Some authors reported no associations between personality features and GD relapse [36]. However, in other studies, neuroticism has been associated with GD relapse [44,45], as have low levels of conscientiousness [44]. High harm-avoidance statistically predicted GD relapse [22], as did high self-transcendence [28]. Persistence may act as a protective factor against relapse in GD [43]. Low levels of self-directedness have been linked to relapse in GD, specifically among individuals with sports betting directedness [37], and high levels of self-directedness may be protective against relapse at GD treatment follow-up [69].

#### 3.2.4. Impulsivity, Executive Functioning, and Relapse

Relationships between impulsivity and relapses in behavioral addictions appear complex. Some GD studies suggested one-year relapses are predicted neither by self-report or behavioral measures of impulsivity or decision-making [70]. Relatedly, self-report measures of both impulsivity and reward sensitivity have failed to statistically predict relapse in individuals with GD [71]. However, high impulsivity and sensation-seeking have been associated with GD relapse [41], as observed with dropout rates, in other studies. More specifically, a dimension of impulsivity frequently associated with relapse is negative urgency. For example, negative urgency statistically predicted the number of relapses during GD treatment among 245 patients who received CBT [46]. Other GD studies linked negative urgency to relapse during treatment [49,53].

Relationships between delay discounting and GD treatment response have also been explored [27]. Among a middle-aged group of individuals with GD, delay discounting was associated with relapse, and steeper discounting statistically predicted relapse [46].

Regarding executive functioning, poor cognitive flexibility has been associated with relapse in follow-up assessments following GD treatment [53]. Moreover, behavioral measures of decision-making (measured by the card-playing task) and disinhibition (measured by the stop-signal reaction time) may predict relapse in individuals with GD [71]. However, other authors have not identified self-reported or behavioral predictors of relapse in GD [51].

#### 3.2.5. Addiction-Related Features, Disorder Severity, and Relapse

Disorder severity. A higher likelihood of relapse may be predicted by lower GD severity [27].

Disorder age of onset/disorder duration. No clear association between age of onset of GD and relapse has been observed [55]. However, a longer duration of GD may predict relapse [71].

Disorder subtypes/preferences. In studies of GD, some authors found no differences in relapses when gambling preferences were considered [36]. However, other studies observed that delay discounting may predict relapse among individuals with GD with preferences for strategic gambling [27].

Other disorder-related features. Gambling-related urges, biases, erroneous cognitions, and distortions have been associated with GD relapse [46,59,72,73]. Specifically, cognitions about winning and feeling a need to make money are associated with GD relapse [74]. Strong cravings may be associated with lower confidence in coping with cravings and, consequently, with relapse [75]. In addition, a higher likelihood of relapse has been observed in women with GD who do not present with gambling-related debts [20]. This finding may reflect that these women may be experiencing less interference from the disorder in their lives and, consequently, may be underestimating negative consequences associated with continued gambling [20]. Having committed gambling-related offenses with legal consequences has also been associated with GD relapse [57]. Other predictors of relapse that have been proposed in the case of GD include spending more than 100 EUR per week on gambling behavior, or any gambling, in part depending on the definition of relapse [22].

#### 3.2.6. Other Factors and Relapse

Other factors associated with relapse include difficulty tolerating boredom and lack of structure during leisure time [59]. Moreover, a protective factor mitigating against relapse appears to be having experienced abstinence for a period of at least one month in the previous year [69]. An association between discrimination, marginalization, and relapse has also been described [76]. Cue-reactivity is another factor that may induce relapse in GD [77]. Finally, behavioral indicators of disinhibition and decision-making may be significant predictors of relapse, whereas reward sensitivity may not [71].

### 3.3. Dropout and Relapse Studies Using Clusters/Trajectories

Some studies explored both dropout and relapse using cluster-based or trajectory analyses. For example, two of four clusters identified in women with GD or CBSD had the poorest treatment outcomes, with more frequent dropout and relapse [78]. These clusters included women with more psychopathology, dysfunctional personality profiles (characterized by high scores on harm avoidance and novelty-seeking and low scores on self-directedness, persistence, and reward-dependence), and self-destructive behavior. Another study that detected clusters taking into account treatment outcome in women with GD identified that one cluster was characterized by the highest likelihood of relapse, GD severity, and levels of psychopathology, elevated harm-avoidance scores, and reduced self-directedness scores [79]. Finally, when exploring response trajectories of GD severity after an intervention for young adults with GD, three distinct trajectories were observed: (a) T1 was composed of individuals with high GD severity at baseline and a good prognosis for recovery; (b) T2 included individuals with moderate-high GD severity at pre-treatment and a good prognosis for recovery; and, (c) T3 was composed of individuals with high GD severity at baseline and poor therapeutic outcomes [80]. It was found that the highest likelihood of poor treatment response was associated with low social status, high emotional distress, high levels of harm-avoidance, and low levels of self-directedness [80].

## 4. Clinical Implications, Limitations, and Future Studies

Behavioral addictions are often characterized by frequent relapse and treatment dropout. A comprehensive study of the factors that precipitate poor adherence to treatment is important for enhancing the development of both prevention and treatment plans that focus on enhancing better treatment responses. As other authors have suggested, reducing the prevalence of behavioral addictions should be promoted by policies and interventions that focus on decreasing the first-time incidence (i.e., new cases) and number of relapsing individuals, as well as on enhancing recovery, prolonging remission, and preventing relapse [65]. Prevention and treatment plans should take into account that age, gender, educational level, and social and marital status, along with certain personality features and the presence of co-occurring disorders, are closely associated with dropout and relapse, and interventions should be targeted to the most vulnerable populations. Additionally, by using instruments that assess specific domains of recovery capital and related constructs such as spirituality, greater insight may be achieved regarding the factors that may be relevant to specific individuals, perhaps promoting personalized medicine approaches.

Across SUDs and behavioral addiction, similar constructs (e.g., assessing recovery capital) have been used to investigate recovery and its underlying mechanisms including in behavioral addictions such as GD [81,82,83,84,85]. Related constructs such as spirituality also warrant consideration [82,83,86,87,88,89]. Similarly, across the disorders, different goals have been used to define recovery (e.g., abstinence in 12 step programs, no longer meeting diagnostic criteria, or falling below a level of hazardous involvement [90,91]). With respect to this last consideration, new entities in the ICD-11 to assess hazardous gambling and hazardous gaming exist [92], analogous, for example, to hazardous alcohol use [2], and the extent to which these constructs may be used to inform recovery in behavioral addictions warrants consideration. Together, there is a need for more research into constructs that may mechanistically underlie recovery, from social, psychological, and particularly neural perspectives. Such research may advance progress towards more personalized approaches to treatment of people with behavioral addictions.

The process of advancing an understanding of recovery from behavioral addictions may best be achieved by addressing the three main limitations of existing studies on relapse and dropout in behavioral addictions. First, it is important to reach a scientific consensus on the definitions of recovery, relapse, and dropout, in order to promote better homogeneity in the assessment of treatment responses and comparability of results. Second, studies with larger sample of the clinical populations than those included in the current literature are required. Third, studies of longer durations are needed. Longer study durations could allow assessments of relapse and dropout at more time points, which would help to better understand these complex phenomena and the longer-term prognoses for people with behavioral addictions. Finally, most existing studies evaluated treatment outcomes only in individuals with GD. More studies exploring dropout and relapse in other behavioral addictions are needed.

## 5. Conclusions

Together, the findings suggest that multiple sociodemographic (mainly age, gender, education level, and employment, social, and civil status) and clinical factors (mainly addiction-related features, co-occurring disorders, and personality features) appear associated with both dropout and relapse in individuals being treated for behavioral addictions, although there are some conflicting results as to whether the associations are positive or negative (see Table 1). However, more consensus regarding definitions of dropout and relapse is required to help ensure greater comparability of results across studies. Such work will help advance the intervention field for behavioral addictions as the etiologies of these behaviors and conditions become better understood.

## Figures and Tables

**Table 1:** Main features included in the present review.

	Dropout	Relapse
Sociodemographic	Age Gender Education level/years of education Employment status Income/social status Civil status and social support	Age Gender Education level/years of education Motherhood Civil status and social support
Co-occurring disorders	Psychopathological distress Suicidality and co-occurring behaviors/disorders	Psychopathological distress Co-occurring behaviors/disorders
Personality features	Conscientiousness Novelty-seeking Self-directedness Cooperativeness Lack of perseverance	Neuroticism Conscientiousness Harm-avoidance Self-transcendence Persistence Self-directedness
Impulsivity and executive functioning	Sensation-seeking Affect-driven impulsivity Reward sensitivity Cognitive flexibility	Sensation-seeking Negative urgency Delay discounting Cognitive flexibility Decision making Disinhibition
Addiction-related features	Disorder severity Disorder age of onset / disorder duration Disorder subtypes/preferences Other disorder-related features	Disorder severity Disorder age of onset / disorder duration Disorder subtypes/preferences Other disorder-related features
Other factors	Emotional regulation Motivation to change	Difficulty tolerating boredom Lack of structure during leisure time Experienced abstinence for a period of at least one month Discrimination and marginalization Cue-reactivity

## Data Availability

Not applicable.
